# Isolated De Winter Pattern in Lead V2: A Faint yet Critical Sign of First Diagonal Artery Occlusion

**DOI:** 10.1111/anec.70115

**Published:** 2025-09-25

**Authors:** Zhong‐Qun Zhan

**Affiliations:** ^1^ Department of Cardiology Shenzhen Guangming District People's Hospital Shenzhen P. R. China

We read with interest the article by Ali Amghaiab et al. titled “Decoding South African Flag Sign—When Lead V2 Speaks Volumes” published in *JAMA Internal Medicine* (Ali Amghaiab et al. 2025). The authors describe a case of suspected ST‐segment elevation myocardial infarction (STEMI) based on isolated ST elevation in lead V2, subtle ST elevation in leads I and aVL, and reciprocal changes in lead III, ultimately attributed to occlusion of the first diagonal (D1) branch of the left anterior descending artery.

Although we commend the authors for highlighting this important and often underrecognized electrocardiographic pattern, we respectfully propose an alternative interpretation of the initial ECG. Upon close inspection, lead V2 does not demonstrate classic ST‐segment elevation. Instead, it exhibits upsloping ST‐segment depression followed by a tall, symmetric T wave, a morphology consistent with the de Winter pattern. Leads I and aVL similarly show hyperacute T waves without definitive ST elevation, suggesting early transmural ischemia rather than established injury current.

The de Winter pattern, originally described in proximal LAD occlusion, is increasingly recognized in isolated D1 occlusion, particularly when the ischemic vector is postero‐inferiorly, aligning with the axis of lead V2 (de Winter et al. 2008). This pattern represents a STEMI equivalent, often preceding overt ST elevation, and mandates urgent reperfusion therapy.

This single‐lead V2 de Winter pattern is, to my knowledge, previously unreported and may represent the earliest electrocardiographic signature of a proximally arising, anatomically dominant D1 branch. Recognition of isolated de Winter morphology in V2 as a solitary anterior lead sign of D1 occlusion is clinically invaluable, especially in the absence of contiguous lead involvement. It expands the spectrum of occlusion myocardial infarction (OMI) patterns and supports the shift from traditional STEMI versus NSTEMI paradigms toward OMI versus NOMI classification (McLaren et al. 2024).

We congratulate the authors on this insightful case and emphasize that hyperacute T waves and de Winter morphology, even in a single lead, should prompt immediate suspicion of coronary occlusion and urgent angiographic evaluation.

## Author Contributions

The author takes full responsibility for this article.

## Conflicts of Interest

The author declares no conflicts of interest.

## Data Availability

The data that support the findings of this study are openly available in http://doi.org/10.1111/anec.70115 at figshare.
